# Identifying Transition to First Episode Psychosis (FEP) From ‘At Risk Mental State’ (ARMS) in Sussex Early Intervention in Psychosis (EIP) Services

**DOI:** 10.1192/bjo.2022.385

**Published:** 2022-06-20

**Authors:** Emma Davies, Richard Whale

**Affiliations:** 1 Sussex Partnership NHS Foundation Trust, Sussex, United Kingdom; 2 Brighton and Sussex Medical School, Brighton, United Kingdom

## Abstract

**Aims:**

Identification of a psychosis risk syndrome to aid reduction of transition to a FEP is an important focus of worldwide research. ARMS for psychosis was defined by Yung and McGorry in 1996. UK EIP services were mandated to identify and ‘treat’ ARMS in the ‘Implementing the Early Intervention in Psychosis Access and Waiting Time Standard: Guidance' 2016. Sussex EIP services developed such an ARMS service with a 1-year pathway of assessment, intervention as indicated, and monitoring from 2017. Sussex serves a population of approximately 1.4 million, including areas with both low and high social deprivation indices. Transition rates from ARMS to FEP in recent studies have suggested widely varying rates of 8–17% of transition in a two-year period, notably less than initially identified by Yung et al. We aimed to establish the rate of transition to FEP within 12 months from identification of ARMS in Sussex EIP services.

**Methods:**

A retrospective study was conducted on all patients on the ARMS pathway, across five EIP services in Sussex, between Jan 2017-Oct 2021. The primary outcome measure was operationally defined transition to FEP; secondary outcome measures included clinical features and use of clinical services.

**Results:**

71 cases were identified as meeting ARMS criteria, with mean age 21.4yo; range 14–35, from a total new caseload of 447 over this period.

ARMS subcategories identified 4 state/trait, 55 attenuated psychosis and 12 BLIPS. Comorbidity was more common than not; mood disorders were identified in 17 cases. 23 cases met not in education, employment or training (NEET) criteria.

All cases received full care coordination by lead practitioners. 19 cases were prescribed atypical antipsychotics. 18 cases received formal CBT.

4 of the 71 cases transitioned to FEP within 12 months at mean time 35 weeks; range 28–45 weeks. 2 had attenuated symptoms and 2 experienced BLIPS. 3 were initially NEET.

**Conclusion:**

We report a very low transition rate to FEP of 6% in this service, consistent with other such UK services. Whilst the ARMS sample is low in number, a clear impact on EIP service case management is identified. Risk saturation is arguably required to justify continuing this ARMS pathway, achievable by primary focus on the BLIPS subgroup. Wider review of UK ARMS services is required to reduce dilution of EIP service models and reduction of their well evidenced effectiveness.

